# Constrictive Pericarditis as a Rare Manifestation of Graft‐Versus‐Host Disease: A Case Report

**DOI:** 10.1002/ccr3.70479

**Published:** 2025-05-02

**Authors:** Alireza Arzhangzadeh, Mahmood Zamirian, Ladan Nasermoadeli, Asma Mousavi, Salma Nozhat, Roozbeh Narimani Javid, Sasan Shafiei, Salar Azadnik, Shayan Shojaei

**Affiliations:** ^1^ Department of Cardiology Shiraz University of Medical Sciences Shiraz Iran; ^2^ Cardiac Primary Prevention Research Center Cardiovascular Diseases Research Institute, Tehran University of Medical Sciences Tehran Iran; ^3^ Tehran Heart Center Cardiovascular Diseases Research Institute, Tehran University of Medical Sciences Tehran Iran; ^4^ Research Center for Advanced Technologies in Cardiovascular Medicine Cardiovascular Diseases Research Institute, Tehran University of Medical Sciences Tehran Iran; ^5^ Department of Internal Medicine Shiraz University of Medical Sciences Shiraz Iran

**Keywords:** allogenic stem cell transplantation, case report, constrictive pericarditis, graft‐versus‐host disease, pericardiectomy

## Abstract

Graft‐versus‐host disease (GVHD) is a serious inflammatory complication that can arise after allogeneic transplantation, characterized by donor T‐cells attacking the recipient's tissues. While cardiac complications are infrequent, they are more commonly observed in cases of chronic GVHD and may manifest as pericardial effusion, cardiac tamponade, and various arrhythmias. Additionally, chronic GVHD can result in constrictive pericarditis (CP) due to the accumulation of fluid and scarring. A 25‐year‐old Iranian man developed CP 14 years after undergoing allogenic stem cell transplantation. Following inadequate response to medical therapy, he underwent a pericardiectomy. Pathological examination during follow‐up revealed fibrosis and mild chronic inflammation. This report aims to add another case of cardiac manifestation associated with GVHD to the current literature. The main message emphasizes the urgent need for prompt diagnosis and effective pericardiectomy, which can be life‐saving. Surgical referral should always be an option, and post‐operative immunomodulation is crucial.

**Abbreviations**
APCs: activate host antigen‐presenting cells
CHB: complete heart block
CP: constrictive pericarditis
CRP: C‐reactive protein
ESR: erythrocyte sedimentation rate
GVHD: graft‐versus‐host disease
HFpEF: heart failure with preserved ejection fraction
HSCT: hematopoietic stem cell transplantation
IFN‐γ: interferon‐gamma
IVC: inferior vena cava
LV: left ventricular
LVEF: left ventricular ejection fraction
NT‐Pro BNP: N‐terminal pro‐B‐type natriuretic peptide
NYHA: New York Heart Association
PE: pericardial effusion
SCD: sudden cardiac death
TNF: tumor necrosis factor
TTE: transthoracic echocardiogram

**Summary**

- Constrictive pericarditis (CP) is an uncommon but serious cardiac complication of chronic graft‐versus‐host disease (GVHD), with a subtle and progressive nature that can make diagnosis challenging.
- Early recognition of CP in post‐transplant patients is crucial. Timely intervention, including pericardiectomy when necessary, can significantly improve quality of life and long‐term outcomes.

## 1 Background

Graft‐versus‐host disease (GVHD) is a severe and often fatal inflammatory complication occurring after transplantation. GVHD is initiated when donor T‐cells launch an immune response against the recipient's tissues, perceiving them as foreign. This process may affect multiple organ systems, including, albeit rarely, the cardiovascular system. Cytokines, including tumor necrosis factor (TNF) and interferon‐gamma (IFN‐γ), activate host antigen‐presenting cells (APCs), which stimulate CD4+ and CD8+ cells, exacerbating inflammation and symptoms [1, 2, 3]. This pathogenic cascade initiates with the upregulation of endothelial adhesion molecules, which facilitates macrophage recruitment for a sustained inflammatory response. Consequently, the inflammatory condition induces fibrinoid necrosis, fibroblast proliferation, and collagen deposition. Collectively, these mechanisms disrupt normal tissue architecture, causing pericardial scars and thickening [4, 5].

In typical immune responses, recipient T‐cells neutralize donor cells. However, due to chemotherapy, radiotherapy, and immunosuppressive medications, recipients often cannot reject transplanted cells [6]. Therefore, this disease is the main cause of delayed morbidity and mortality following allogeneic hematopoietic stem cell transplantation (HSCT). The condition demonstrates various clinical presentations, ranging from localized cutaneous involvement to life‐threatening multiorgan dysfunction. Besides corticosteroids, there is no other recommended therapy for this condition [7, 8].

While cardiac involvement in GVHD is uncommon, it tends to occur more often in the chronic form compared to the acute form [9]. The cardiac manifestations of chronic GVHD are diverse and can include pericardial effusion (PE) [10, 11, 12], cardiac tamponade [12, 13, 14], cardiogenic shock [15], mitral regurgitation [15], bradycardia [16], and ventricular arrhythmias including ventricular fibrillation [17, 18], complete heart block (CHB) [19] and sudden cardiac death (SCD) [20]. Studies on echocardiographic findings in chronic GVHD primarily focus on left ventricular systolic function, which is often compromised by the cardiotoxic effects of pre‐transplant chemotherapy, reducing left ventricular ejection fraction (LVEF) [21, 22].

Constriction of the pericardium, leading to constrictive pericarditis (CP), may occur due to pericardial scarring and rigidity, especially when pericardial fluid accumulation exceeds the pericardial reserve capacity or fibrotic changes make the pericardium inelastic [23]. Diagnosis of CP relies on imaging, particularly echocardiography, which provides insights into cardiac physiology and pericardial thickening. Accurate imaging is essential, as some underlying causes of CP may be reversible with timely treatment [24].

CP is an uncommon manifestation of chronic GVHD and typically appears as a delayed complication, presenting from three to 30 months post‐transplant depending on patient history and etiology. While not all patients require pericardiectomy, some respond well to medication with favorable prognoses [25, 26, 27, 28].

In this report, we present the case of a 25‐year‐old male who developed CP 14 years after an allogeneic stem cell transplant. You can find the CARE checklist regarding this study in Figure S1.

## 2 Case Presentation

**FIGURE 1:**
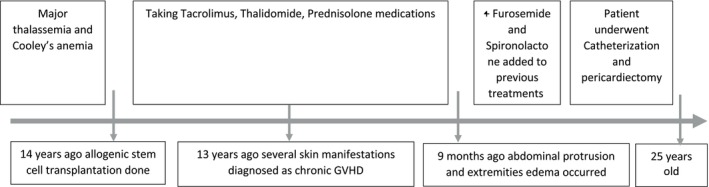
Timeline of our reported patient.

The patient is a 25‐year‐old Iranian male with major thalassemia and Cooley's anemia. 14 years prior, he underwent allogeneic stem cell transplantation due to severe morbidity associated with these conditions, with his mother serving as the donor. Subsequently, he was treated with Tacrolimus, Thalidomide, and Prednisolone as immunosuppressive therapy to prevent graft rejection post‐transplantation (Figure 1).

**FIGURE 2:**
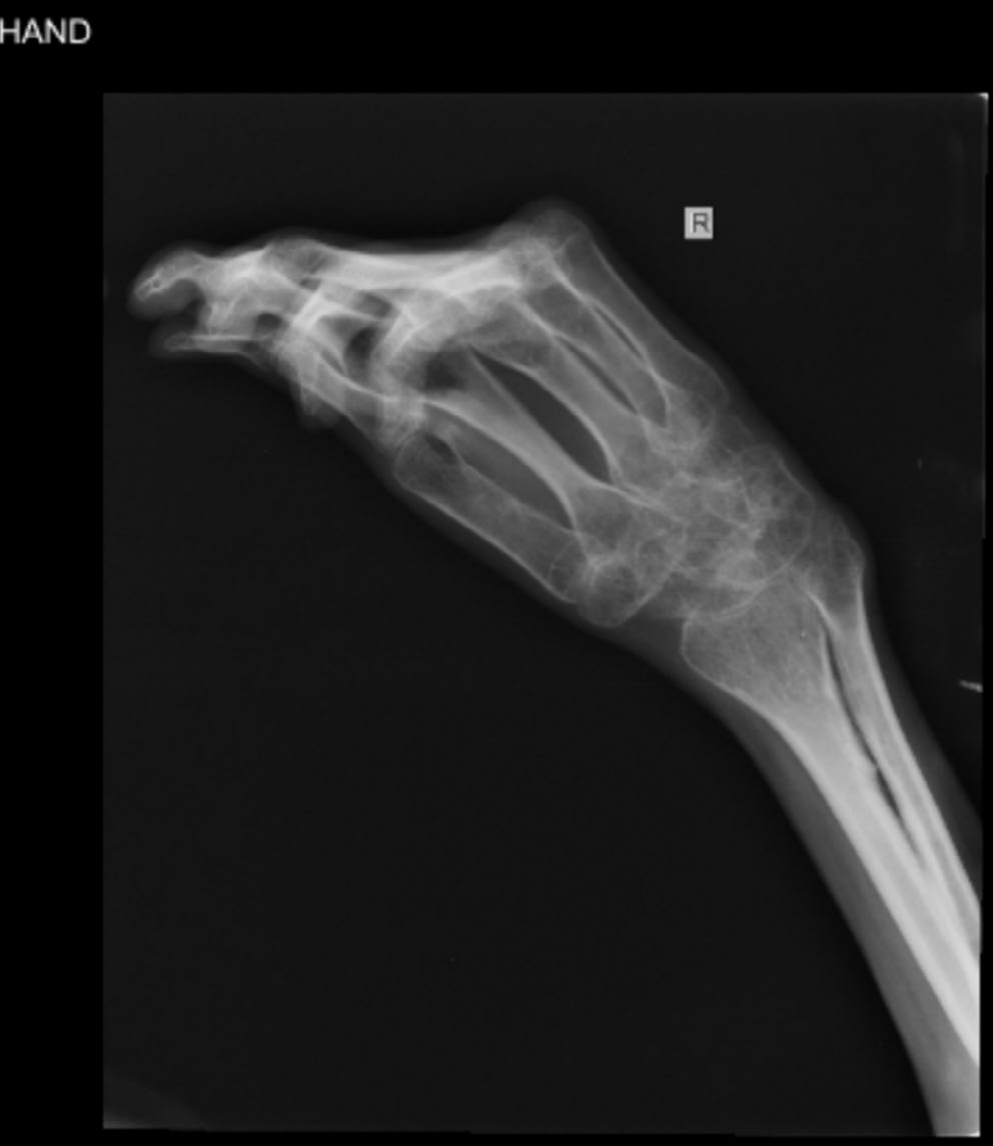
X‐ray of right hand. Musculoskeletal deformities in upper limb.

1 year following the transplant, the patient developed dermatological manifestations diagnosed as chronic GVHD. Musculoskeletal deformities, particularly in the upper limbs, as well as the New York Heart Association (NYHA) class III, were also observed. These symptoms led to significant functional limitations (Figure 2). Treatment involved systemic medications, which were discontinued after symptom resolution. The patient remained largely asymptomatic until 9 months ago, when he presented with abdominal protrusion and edema in the extremities.

### 2.1 Clinical Examination and Initial Investigations

On examination, the patient's heart rate was 98 bpm, blood pressure 100/70 mmHg, with elevated jugular venous pressure. Lung auscultation revealed decreased breath sounds on the right side, indicative of unilateral pleural effusion. No pericardial knock was detected on cardiac auscultation; however, Kussmaul's sign was observed with deep respiration. Physical examination noted abdominal distention and bilateral lower extremity pitting edema (+3 intensity). Baseline electrocardiography showed only sinus tachycardia.

Routine laboratory tests, including blood cell counts, kidney, thyroid, and liver function, were within normal limits. N‐terminal pro‐B‐type natriuretic peptide (NT‐Pro BNP) levels were significantly elevated, indicating a cardiac etiology for his dyspnea, which could be due to different cardiac diseases, such as heart failure, valvular heart disease, or CP. C‐reactive protein (CRP) and erythrocyte sedimentation rate (ESR) were mildly elevated, while the rheumatological panel was unremarkable.

**FIGURE 3:**
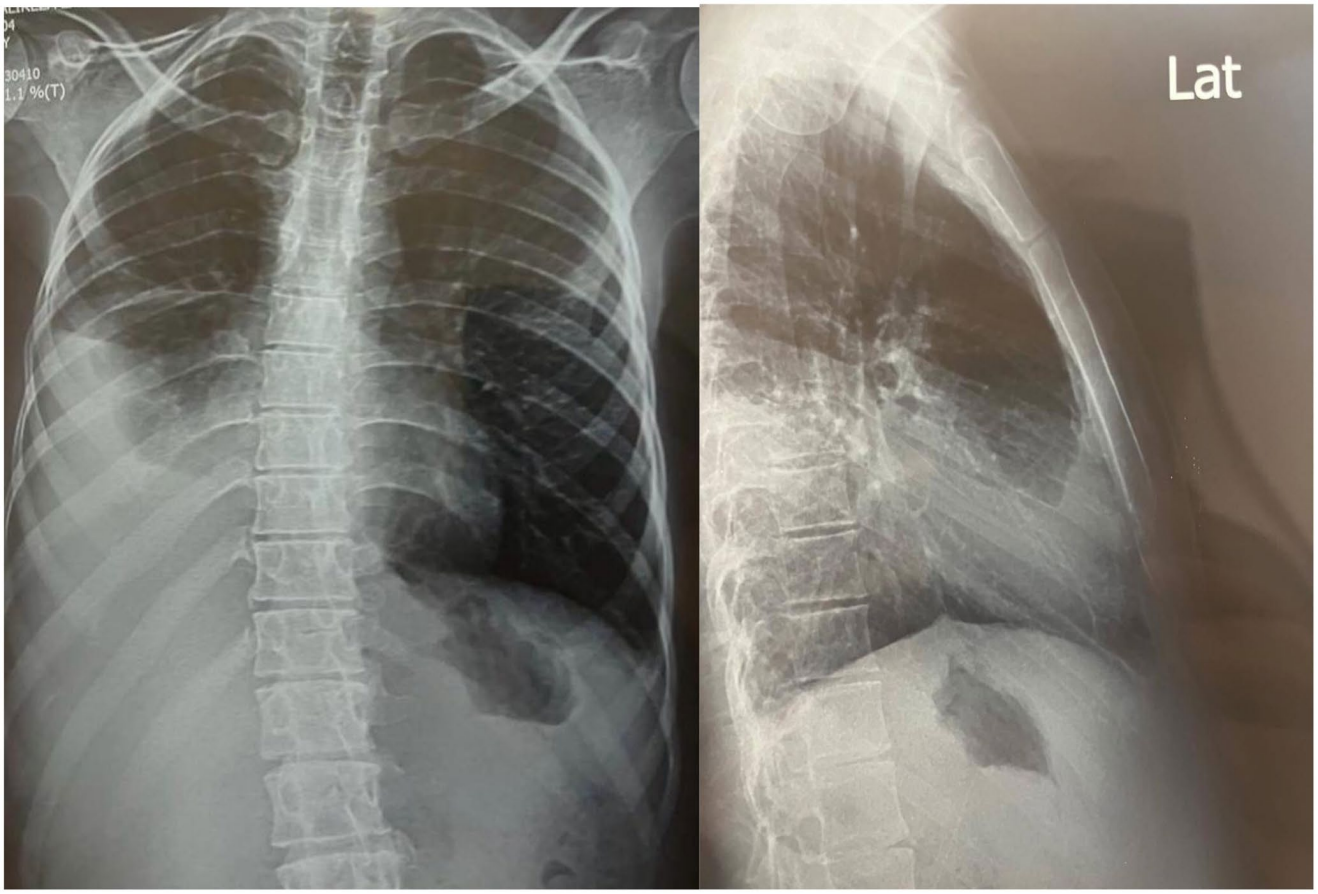
Posterior‐to‐anterior and lateral chest X‐rays. Substantial unilateral pleural effusion was seen.

Chest X‐ray showed a substantial unilateral pleural effusion. Abdominal ultrasound revealed ascites with transudative fluid characteristics (Figure 3).

### 2.2 Diagnosis and Treatment

Due to progressive dyspnea, a transthoracic echocardiogram (TTE) was performed, revealing CP. Prior echocardiograms showed no evidence of CP, indicating its recent development. Echocardiography findings included preserved left ventricular (LV) systolic function with an ejection fraction of 56%, advanced diastolic dysfunction with elevated filling pressures, a right ventricular (RV) basal diameter of 38 mm, and preserved RV function (TAPSE: 19 mm; RV peak systolic myocardial velocity: 9.3 cm/s). Right atrial enlargement (volume index: 34 mL/m^2^) and borderline left atrial enlargement (volume index: 33 mL/m^2^) were noted.

Echocardiography revealed features indicative of constrictive pericarditis (CP), including pericardial thickening, a dilated inferior vena cava (IVC) without collapse, abnormal ventricular septal motion, respiratory septal shudder, and diastolic reversal in the hepatic veins during expiration. There was also prominent respiratory variation in mitral E‐wave velocity and preserved longitudinal systolic strain with reduced strain of the lateral compared to the septal wall (average GLS: −18.7%, lateral wall: −14.7%, septal wall: −19.1%) (Table 1).

**TABLE 1:** Summary of echocardiography data.

LV	RV	LA	RA	Mitral inflow TDI	Speckle tracking findings	Special characteristics of CP
Preserved LV systolic function	Preserved RV function	Top normal LA size and Ap diameter of 37 mm	Enlargement of RA	Prominent respiratory variation in mitral E‐wave velocity	Average GLS −18.7%	Thickening of pericardium
LVEF = 56%	Top normal size RV with basal diameter of 38 mm	LA volume index of 33 mL/m^2^	RA volume index of 34 mL/m^2^	Preserved mitral medial E‐wave velocity (10.9 cm/s)	Reduced LS of lateral wall −14.7% in comparison with septal wall −19.1%	Ventricular septal shudder Abnormal ventricular septal motion
Advanced diastolic dysfunction with elevated filling pressure	TAPSE = 19 mm and RVSm = 9.3 cm/s					Plethora of IVC Hepatic vein diastolic reversal

Abbreviations: CP, constrictive pericarditis; GLS, global longitudinal strain; IVC, inferior vena cava; LA, left atrium; LV, left ventricle; LVEF, left ventricle ejection fraction; RA, right atrium; RV, right ventricle; RVSm, right ventricular peak systolic myocardial velocity; TAPSE, tricuspid annular plane systolic excursion; TDI, tissue doppler echocardiography.

Although cardiac CT and MRI are helpful in diagnosing CP, the patient's severe elbow contractures and the clear echocardiographic findings made these additional imaging techniques unnecessary. These unnecessary modalities could be a significant burden on patients, regarding financial and emotional status. Moreover, CT scans and MRI are conducted by professional, expensive equipment where some institutions lack them. Additionally, the patient's aforementioned musculoskeletal contractures imposed significant positional constraints, which inhibit us from utilizing CMR due to the patient's inability to lie flat or remain still.

### 2.3 Key Factors Supporting GVHD‐Related Constrictive Pericarditis

1. A temporal relationship was observed between the onset of pericardial symptoms and prior transplantation, consistent with GVHD pathophysiology. Moreover, the incidence of preceding events which were not seen in idiopathic pericarditis, coupled with the low incidence rate of the idiopathic type, further supported GVHD as the underlying etiology [29].

In order to unify the patient's clinical presentation, the Systemic involvement pattern in several organs also aligned with established diagnostic criteria for advanced‐stage GVHD [7].

Lymphocytic infiltration and fibrosis in the histopathological examination, which are hallmark features of GVHD [30].

2 A rigorous diagnostic workup excluded alternative etiologies. For instance, we utilized tuberculosis, bacterial, fungal, and viral serologies for infectious causes, which all showed no evidence of microbial pathogens. We also examined pericardial fluid culture post‐surgery, which was negative for all aforementioned markers. Moreover, autoantibodies for various rheumatological conditions, such as systemic lupus erythematosus (SLE), rheumatoid arthritis (RA), and sjogren's syndrome, were also negative.

### 2.4 Hemodynamic Assessment

Following diagnosis, the patient was initially prescribed Furosemide and Spironolactone. However, these diuretics were temporarily discontinued prior to cardiac catheterization to avoid interfering with hemodynamic measurements. Moreover, the patient was started on a regimen of Prednisolone at a dose of 0.5 mg/kg/day for several weeks in order to suppress the immune system. Subsequently, treatment was escalated to include a regimen incorporating calcineurin inhibitors (cyclosporin). Catheterization findings confirmed CP, showing elevated and equalized right and left ventricular diastolic pressures with a characteristic dip‐and‐plateau waveform. Other indicators included right atrial blood flow augmentation during inspiration (Kussmaul's sign), end‐diastolic pressure equalization, pulmonary arterial pressure below 55 mmHg (38 mmHg in this case), RV end‐diastolic pressure > 1/3 of RV systolic pressure, and an LV rapid filling wave height > 7 mmHg (11 mmHg in this case).

**FIGURE 4:**
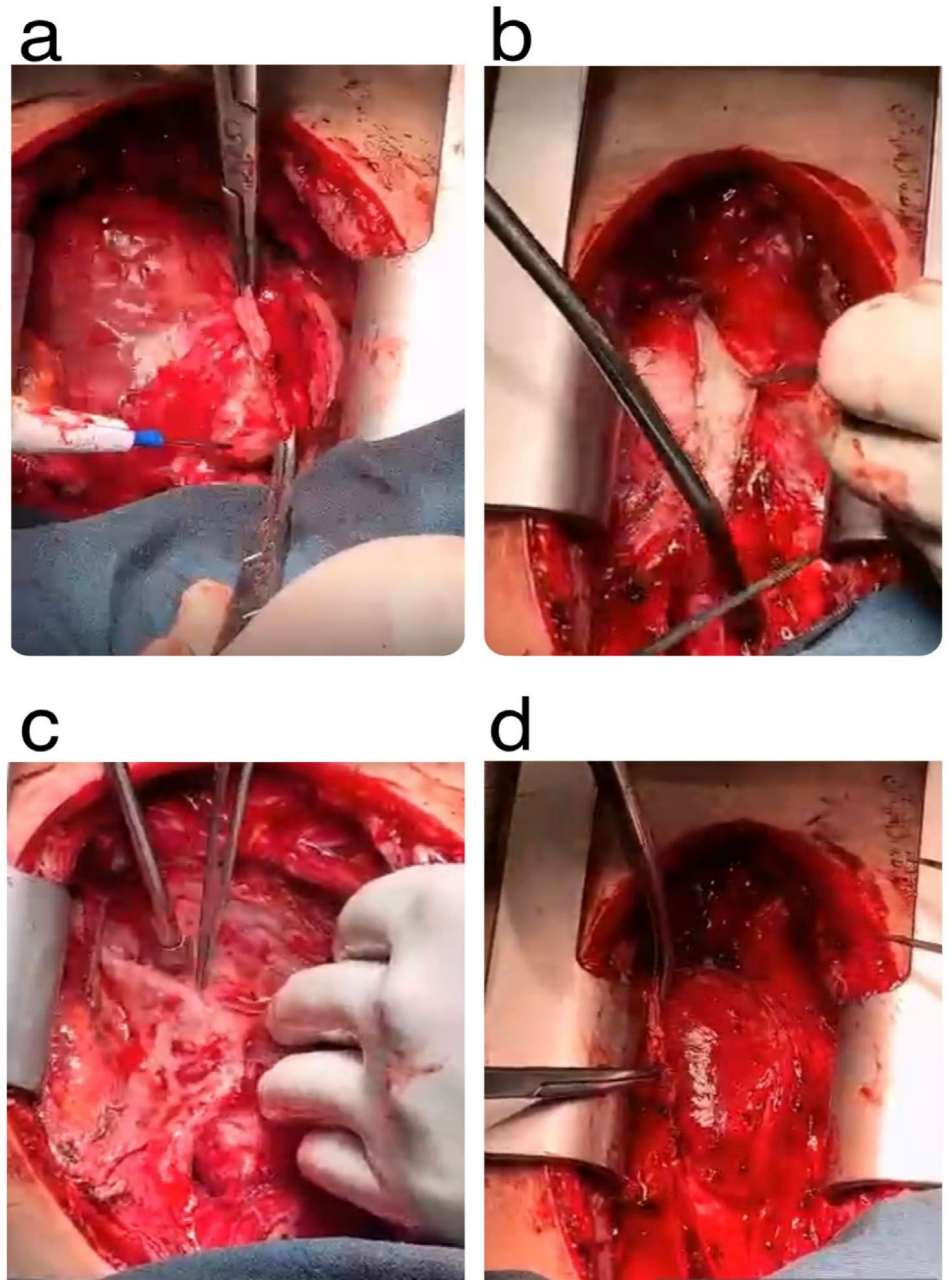
Surgery images. (a) Left pericardial release over the left ventricle. (b) Anterior pericardial release to demonstrating the thickness and fibrosis of the pericardium. (c) Releasing the residual right constricted pericardium with a thickness of 10 mm. (d) Releasing the pericardium around atrial inflow and ventricular outflow tracts for optimal result.

Despite initial diuretic therapy and salt restriction, the patient's volume overload symptoms persisted. Additionally, we leveraged the anti‐inflammatory properties of corticosteroids and T‐cell inhibitory effects of calcineurin inhibitor in our therapeutic strategy. Despite the patient's adherence to the treatment regimen, there was no observable improvement in his clinical condition. Given the variability in patient responses to pharmacological therapy and the potential for inadequate outcomes, a multidisciplinary approach was adopted. Consequently, he underwent pericardiectomy through median sternotomy, with complete pericardium removal (Figure 4).

### 2.5 Outcome and Follow‐Up

The patient showed significant symptomatic and hemodynamic improvement following pericardiectomy. Postoperative echocardiography demonstrated reduced respiratory variation in both ventricles, near‐normal LV diastolic function (Grade 1 LVDD), and an improvement in NYHA functional class from IV to II. These improvements suggested near‐normal ventricular filling patterns and improved hemodynamics post‐pericardiectomy. Pathological examination of the excised pericardium revealed fibrosis and mild chronic inflammation consistent with chronic GVHD. After the treatment, the patient underwent the Minnesota living with heart failure questionnaire (MLHFQ) where his scores demonstrated significant improvement in the quality of life. Our patient adhered to the therapy, which was evaluated by follow‐up calls. Moreover, he has attended each follow‐up session.

## 3 Discussion and Conclusions

CP is a rare etiology of heart failure with preserved ejection fraction (HFpEF) that presents with a complex clinical course. Typically, CP is diagnosed at the initial presentation, with the subsequent challenge of determining the underlying cause. This case report describes an unusual and reversible cause of HFpEF in a patient with chronic GVHD.

Allogeneic hematopoietic stem cell transplantation (HSCT) remains a cornerstone treatment for certain hematologic malignancies. The immune dynamics of allogeneic HSCT are complex, involving potential immune interactions between donor and recipient cells [31]. The pathophysiology of GVHD‐related cardiac involvement parallels that in other organs: immune‐driven inflammation by donor cells leads to fibrosis and cardiac dysfunction. Rackley et al. documented cardiac manifestations of GVHD in 11 patients, predominantly bradyarrhythmias and sudden cardiac death [16]. Nevertheless, reports of pericardial involvement remain rare. Histopathology of this patient's pericardium showed fibrous tissue with lymphocytic infiltration, characteristic of chronic GVHD. The chronic IFN‐γ exposure shifts tissue repair mechanisms toward aberrant extracellular matrix remodeling, which results in fibrotic constriction [32]. Table 2 demonstrates similar cases with a history of transplantation and GVHD with cardiac manifestations.

**TABLE 2:** Similar cases with a history of transplantation and GVHD with cardiac manifestations.

Gender	Age	Disease	Donor	GVHD prophylaxis	Cardiac manifestation	Time after transplantation
Female [10]	10 years	Relapsed ALL	Allogenic bone marrow transplantation from her HLA‐matched sibling	Prednisolone, cyclosporin, methylprednisolone, tacrolimus, methotrexate	Massive pericardial/pleural effusion with anasarca	216 days
Male [11]	29 years	CML	An allogeneic bone marrow transplantation from an HLA‐identical sibling	Methotrexate and cyclosporine	Heart failure, left ventricular wall thickening, left ventricular restrictive filling pattern, pericardial effusion	55 days
Male [12]	62 years	AML	Allogenic hematopoietic stem cell transplantation	Steroid therapy	Pericardial effusion, low cardiac output, and cardiac tamponade	7 days
Male [13]	39 years	AML M2	Allogenic hematopoietic stem cell transplantation from a female HLA‐identical‐related donor	Cyclosporine and methotrexate	Cardiac tamponade	16 months
Male [14]	13 years	CML	Allogeneic BMT from an HLA‐identical unrelated donor	Prednisolone, methotrexate, and cyclosporine	Globular cardiac silhouette, faint heart sounds, pericardial effusion, cardiac tamponade	249 days
[16]	18 months	Wiskott‐Aldrich syndrome	Matched unrelated donor cord blood	Methylprednisolone and cyclosporine	Bradycardia	1 week
[16]	21 months	Baller‐Gerold syndrome, Combined immunodeficiency (Omenn syndrome‐like)	Matched unrelated donor cord blood	Methylprednisolone and cyclosporine	Bradycardia	17 months
[16]	13 years	Refractory large‐cell lymphoma	Peripheral blood stem cell transplantation from a matched sibling donor	Cyclosporine and methotrexate	Bradycardia	1.5 months
[16]	12 years	ALL	Haploidentical peripheral blood stem cell transplantation from mother	T‐cell depletion	Bradycardia	4 months
[16]	13 years	MDS, AML, untreated	Haploidentical T cell‐depleted bone marrow from father	T‐cell depletion	Bradycardia	6.5 months
Male [17]	44 months	Chronic granulomatosis disease	Allogeneic HSCT using peripheral blood stem cells from a human leukocyte antigen‐matched unrelated donor	No prophylaxis	Ventricular fibrillation, mild left ventricular dilatation with hypokinesia, ST changes in electrocardiography, myocardial thinning, narrowing of LAD, and luminal irregularity in RCA and LCX	31 months
Male [18]	17 years	AML M4	Peripheral blood stem cell transplantation from an HLA‐identical unrelated donor	No prophylaxis	Global dysfunction with an ejection fraction of less than 30% and small pericardial effusion, sinus tachycardia, acute ventricular fibrillation, and cardiopulmonary resuscitation	15 days
Male [19]	4 months	Severe combined immunodeficiency	Haploidentical T cell‐depleted bone marrow from his father	No prophylaxis	Decreased cardiac output, bradycardia, junctional rhythm, intermittent second‐degree heart block, complete atrioventricular block, and asystole episodes	8 days
Male [20]	25 months	Wiskott‐Aldrich syndrome (combined immunodeficiency)	Bone marrow transplant using a female unrelated donor matched at A, B, and DR	Anti‐thymocyte globulin, cyclosporin, prednisolone	Heart enlarged weighing, pulmonary and mitral valves dilated, aortic and tricuspid valves slightly wider than expected, inner half of the left ventricular myocardium was abnormally pale with mottling of the outer half of the wall, rapid narrowing of the vessels with severe to near total obliteration, sudden cardiac death	18.5 months
Male [33]	38 years	ALL	Hematopoietic stem cell transplant from his human leukocyte antigen identical sister	Cyclosporin and mycophenolate mofetil	Myocarditis	12 years
Female [34]	19 years	Refractory leukemia	Allogeneic bone marrow transplantation	Methotrexate	Severe coronary artery disease and acute myocardial infarction	30 months

Abbreviations: ALL, acute lymphoblastic leukemia; AML, acute myeloblastic leukemia; CML, chronic myelogenous leukemia; MDS, myelodysplastic syndrome.

The precious follow‐up plan, various modalities used in the diagnosis process, and precise history of our patient empower the reported data in our study. However, the lack of long‐term follow‐ups and utilization of telephone calls for evaluating the adherence of our patients could affect the availability of our presentation. This case underscores the importance of targeted immunotherapy and systemic immunomodulation in managing chronic GVHD, with a multidisciplinary approach involving hematologists. The goal of this report is to expand the literature on CP as a manifestation of GVHD and to highlight the critical role of prompt diagnosis and pericardiectomy, both of which are life‐changing and potentially life‐saving. Surgical referral should not be delayed, and postoperative immunomodulation is essential.

## Author Contributions

**Alireza Arzhangzadeh:** conceptualization, investigation, supervision, validation, writing – review and editing. **Mahmood Zamirian:** supervision, validation, writing – review and editing. **Ladan Nasermoadeli:** investigation, validation, writing – original draft. **Asma Mousavi:** investigation, methodology, writing – original draft. **Salma Nozhat:** conceptualization, writing – original draft. **Roozbeh Narimani Javid:** conceptualization, methodology. **Sasan Shafiei:** conceptualization, investigation, methodology. **Salar Azadnik:** conceptualization, investigation. **Shayan Shojaei:** conceptualization, validation, writing – original draft.

## Ethics Statement

We conducted following the principles outlined in the Declaration of Helsinki.

## Consent

We have obtained written informed consent from the patient to publish this report in accordance with the journal's patient consent policy.

## Conflicts of Interest

The authors declare no conflicts of interest.

## Supporting information


Figure S1.

## Data Availability

The data utilized in this study can be obtained from the corresponding author upon reasonable request.
