# Supplementary material for: Constrictive Pericarditis as a Rare Manifestation of Graft‐Versus‐Host Disease: A Case Report
Source: Clin Case Rep. 2025 May 2;13(5):e70479. doi: 10.1002/ccr3.70479 (PMC12048705; doi:10.1002/ccr3.70479)
Supplement: Supplementary file 1 — Figure S1. [file CCR3-13-e70479-s001.pdf]

CARE
case report guidelines

CARE Checklist of information to include when writing a case report

(cc) BY-NC-ND

Topic	Item	Checklist item description	Reported on Line
**Title**	1	The diagnosis or intervention of primary focus followed by the words "case report"	1-2
**Key Words**	2	2 to 5 key words that identify diagnoses or interventions in this case report, including "case report"	44
**Abstract (no references)**	3a	Introduction: What is unique about this case and what does it add to the scientific literature?	28-33
	3b	Main symptoms and/or important clinical findings	34-35
	3c	The main diagnoses, therapeutic interventions, and outcomes	35-37
	3d	Conclusion—What is the main "take-away" lesson(s) from this case?	38-41
**Introduction**	4	One or two paragraphs summarizing why this case is unique (**may include references**)	47-75
**Patient Information**	5a	De-identified patient specific information.	84-88
	5b	Primary concerns and symptoms of the patient.	90-95
	5c	Medical, family, and psycho-social history including relevant genetic information	90-92
	5d	Relevant past interventions with outcomes	92-93
**Clinical Findings**	6	Describe significant physical examination (PE) and important clinical findings.	99-104
**Timeline**	7	Historical and current information from this episode of care organized as a timeline	81
**Diagnostic Assessment**	8a	Diagnostic testing (such as PE, laboratory testing, imaging, surveys).	104-150
	8b	Diagnostic challenges (such as access to testing, financial, or cultural)	132-134
	8c	Diagnosis (including other diagnoses considered)	107-108
	8d	Prognosis (such as staging in oncology) where applicable	
**Therapeutic Intervention**	9a	Types of therapeutic intervention (such as pharmacologic, surgical, preventive, self-care)	145
	9b	Administration of therapeutic intervention (such as dosage, strength, duration)	146-153
	9c	Changes in therapeutic intervention (with rationale)	156-157
**Follow-up and Outcomes**	10a	Clinician and patient-assessed outcomes (if available)	160-161
	10b	Important follow-up diagnostic and other test results	161-164
	10c	Intervention adherence and tolerability (How was this assessed?)	164-166
	10d	Adverse and unanticipated events	-
**Discussion**	11a	A scientific discussion of the strengths AND limitations associated with this case report	173-176 , 186-189
	11b	Discussion of the relevant medical literature **with references**.	177-185
	11c	The scientific rationale for any conclusions (including assessment of possible causes)	189-191
	11d	The primary "take-away" lessons of this case report (without references) in a one paragraph conclusion	191-194
**Patient Perspective**	12	The patient should share their perspective in one to two paragraphs on the treatment(s) they received	197-199
**Informed Consent**	13	Did the patient give informed consent? Please provide if requested	**Yes** ☐ yes **No** ☐
